# Decreased expression of semaphorin 3D is associated with genesis and development in colorectal cancer

**DOI:** 10.1186/s12957-017-1128-1

**Published:** 2017-03-20

**Authors:** Zhen Wang, Meiman Ding, Naiying Qian, Beifeng Song, Jiayin Yu, Jinlong Tang, Jingyu Wang

**Affiliations:** 1 Department of Pathology, The First Hospital of Jiaxing, Zhejiang, People’s Republic of China; 2 The Criminal Investigation Detachment of Jiaxing Public Security Bureau, Zhejiang, People’s Republic of China; 3 Department of Pathology, Second Affiliated Hospital, Zhejiang University School of Medicine, Hangzhou, People’s Republic of China

**Keywords:** SEMA3D, Colorectal cancer, Metastasis, Prognosis

## Abstract

**Background:**

Semaphorin 3D (SEMA3D) plays important roles in the genesis and progress of many cancers. However, the relationship between SEMA3D and colorectal cancer (CRC) remains unknown. The aim of this study was to investigate whether SEMA3D can be used as a predictive marker for the diagnosis, metastasis, and prognosis of CRC by assessing the expression of SEMA3D in the tissues and serum of CRC patients.

**Methods:**

Real-time quantitative polymerase chain reaction (qPCR) was used to measure the expression of SEMA3D mRNA in 100 CRC tissues and matched normal tissues. qPCR was also used to detect the expression of SEMA3D mRNA in the CRC cell line RKO. RKO cells were transfected with SEMA3D small-interring RNA (siRNA) to interfere with endogenous SEMA3D. The migratory ability of control and SEMA3D siRNA-transfected RKO cells was determined by transwell assays. Enzyme-linked immunosorbent assay (ELISA) was utilized to detect the levels of SEMA3D in the serum of 80 CRC patients and 100 normal healthy controls. The expression of SEMA3D in 215 CRC tissues was assessed using immunohistochemistry (IHC). Then, statistical analyses were adopted to assess SEMA3D protein levels and clinical pathological characteristics.

**Results:**

The mRNA expression of SEMA3D was significantly lower in CRC tissues than in paired normal tissues (*t* = 5.027, *P* < 0.0001). Compared with normal healthy controls, the serum levels of SEMA3D were decreased significantly in CRC patients (*t* = 3.656, *P* = 0.0003). The expression of SEMA3D protein was linked to lymph node metastasis, and low expression led to lymph node metastasis (*χ* ^2^ = 8.415*, P* = 0.004). The expression of SEMA3D in CRC tissues was a favorable prognostic factor. Patients with a higher expression of SEMA3D experienced longer survival (*P* = 0.002, log-rank [Mantel-Cox]; Kaplan-Meier). In addition, multivariate Cox’s proportional hazard model revealed that SEMA3D is an independent prognostic marker (hazard ratio [HR] 1.818, 95% CI 1.063–3.110, *P* = 0.029). Moreover, transwell assays showed that knocking down SEMA3D significantly increased RKO cell migration (*t* = 9.268, *P* = 0.0008).

**Conclusions:**

SEMA3D might function as a tumor suppressor during the formation and development of CRC. SEMA3D might become a predictive marker for the diagnosis, metastasis, and prognosis of CRC and provide a novel target for the prevention and treatment of CRC.

## Background

Colorectal cancer is a common malignant tumor occuring in the digestive system and affects patients worldwide. The International Agency for Research on Cancer (IARC) reported that the worldwide new incidence rate of CRC was the third and second highest cancer in males and females, respectively, in 2012. However, CRC had the fourth and third highest cancer-associated mortality rate worldwide in males and females, respectively [1]. Recently, a Chinese study revealed that 376,300 new cases of CRC and 191,000 fatalities were expected in 2015. In new cases, men and women are accounted for fifth and fourth, respectively. However, in mortality cases, both men and women are accounted for fifth [2]. These authoritative data suggest that CRC remains a major threat to human health. The development of CRC is associated with complex interactions between multiple genes and signaling pathways. However, the mechanism behind CRC is still not fully understood. In addition, tumor metastasis is an important contributor to mortality in patients with CRC [3].

Recent research showed that SEMA3D which encodes a member of the semaphorin III family of secreted signaling proteins that are involved in axon guidance during neuronal development has a certain correlation with the development of breast cancer, glioblastoma, pancreatic cancer, thyroid cancer, and other tumors [4–8]. However, no link between SEMA3D and CRC has been reported.

Our previous study used mRNA expression profile microarrays to show that the expression of SEMA3D was significantly lower in CRC tissues than paired normal tissues. Based on these results, the current study further detected the mRNA and protein expression of SEMA3D in CRC and paired normal tissues. Furthermore, the serum levels of SEMA3D were measured in CRC patients and normal healthy controls. This study discussed the role of SEMA3D in CRC, and the results may provide an experimental basis for further research into the formation, development, prevention, diagnosis, and individualized treatment of CRC.

## Methods

### Patients and specimens

CRC tissue specimens and paired normal mucosa for mRNA detection were available from 100 CRC patients (57 males and 43 females aged 34–86 years old, mean age ± standard deviation (SD) 64.9 ± 11.4 years) who underwent surgery between January and December 2015 at the First Hospital of Jiaxing. All the tissues were frozen in liquid nitrogen immediately after surgery and then stored at −80 °C until RNA extraction. A total of 215 formalin-fixed, paraffin-embedded CRC tissues (134 male and 81 female patients aged 30–86 years, old, mean age ± SD, 63.8 ± 11.0 years) from patients who were recruited from the First Hospital of Jiaxing between January 2009 and December 2010 were used for immunohistochemistry. All these cases were followed-up, and clinicopathological data were collected from medical records. In addition, 80 serum samples from CRC patients (42 males and 38 females aged 28–86 years; mean age ± SD, 65.1 ± 11.7 years) and 100 serum samples from normal healthy control subjects (52 males and 48 females aged 28–82 years, mean age ± SD 61.6 ± 9.1 years) were obtained from the First Hospital of Jiaxing between January and December 2015. All the blood samples were centrifuged at 4 °C, aliquoted, and stored at −80 °C until analysis by ELISA.

All samples from CRC patients were harvested before radiotherapy or chemotherapy. All samples underwent surgery, and all cases were diagnosed by two pathologists independently.

### Real-time quantitative polymerase chain reaction (qPCR)

Total RNA was extracted from tissues using an RNeasy Mini Kit (Qiagen, Catalog no. 74106) and cDNA was synthesized using a PrimeScript^®^ RT reagent kit (TaKaRa Biotechnology, Dalian, China, Catalog no. RR037A). Q-PCR was performed using SYBR® Premix Ex Taq (TaKaRa; Catalog no. RR420A), and the relative gene expression levels were calculated using the 2^−∆∆Ct^ method; glyceraldehyde-3-phosphate dehydrogenase (GAPDH) was used as an internal control. The primer sequences used were as follows: GAPDH forward, 5’-ACCACAGTCCATGCCATCAC-3’and reverse, 5’-TCCACCACCCTGTTGCTGTA-3’ (product length, 452 bp); SEMA3D forward, 5’-TGGGACATCGAAGACAGCAT-3’ and reverse, 5’-AAAGTGTGCTCCTGGGCTTT-3’ (product length, 263 bp).

### Immunohistochemistry

Formalin-fixed, paraffin-embedded sections of all 215 tissues were subjected to the EnVision method using SEMA3D polyclonal antibodies (Novus, American, catalog no. NBP1-85517, 1:50). Phosphate-buffered Saline (PBS) was used as a negative control instead of the primary antibody. Sections were counterstained with hematoxylin.

### Scoring method

As shown in Table 1, the total SEMA3D immunostaining was scored as both the percentage of positive cells and the intensity of the cytoplasmic/cytomembrane staining. All sections were reviewed by two experienced pathologists independently. The SEMA3D staining score of each section was calculated by multiplying the percentage and the intensity scores. A minimum measurement score of 6 was defined as the overexpression of SEMA3D, whereas a score < 6 was regarded as low expression.

**Table 1:** The scoring criteria of immunohistochemistry

The percentage of positive tumor cells	Score	The intensity of staining	Score
0	0	No staining	0
<10%	1	+	1
10–50%	2	++	2
51–80%	3	+++	3
>80%	4		

### ELISA

The serum concentrations of SEMA3D were measured using a sandwich enzyme-linked immunosorbent assay kit according to the manufacturer’s instructions (Cusabio, China, catalog no. CSB-EL020983HU).

### Cell culture and siRNA transfection

The human CRC line RKO was purchased from the American Type Culture Collection (Manassas, VA, USA), was maintained in RPMI 1640 medium supplemented with 10% fetal bovine serum (HyClone, Tauranga, New Zealand), and was grown at 37 °C in an atmosphere containing 95% air and 5% CO_2._ siRNA specific for SEMA3D, and negative control were purchased from Genepharma (Shanghai, China). All transfection experiments were performed using Lipofectamine 2000 (Invitrogen), incubated for 48 h according to the manufacturer’s instructions.

### Cell migration

To evaluate the migratory capacity of RKO cells, 24-well plates equipped with cell culture inserts containing 8.0 μm pore size membranes (Costar Corp., Cambridge, MA, USA) were used. Briefly, a total of 5 × 10^4^ cells were resuspended in 100 μl of serum-free medium and placed in the upper chambers. The lower chamber was filled with 10% PBS as the chemoattractant. At the end of the experiments, cells on the upper surface of the filters were removed, and cells that had migrated to the lower surface were fixed in 4% paraformaldehyde and stained with 0.1% crystal violet. Then, the crystal violet was quantified at 570 nm using spectrophotometry after elution in 33% glacial acetic acid.

### Statistical analysis

SPSS19.0 was utilized for all statistical analyses. Measurement data are presented as means ± standard deviations ($\overline{\mathrm{x}}$±s) and were compared using paired or unpaired *t* tests as appropriate. Chi-square tests were used to analyze the IHC data to identify correlations between clinicopathological parameters and SEMA3D protein levels. Overall survival was evaluated using log-rank tests, and survival curves were plotted according to the Kaplan-Meier method. Prognostic variables were analyzed using the multivariate Cox’s proportional hazard model. All statistics were two-sided. *P* values <0.05 were considered to indicate statistical significance.

## Results

### SEMA3D is differentially expressed between CRC and paired normal tissues

**Fig. 1:**
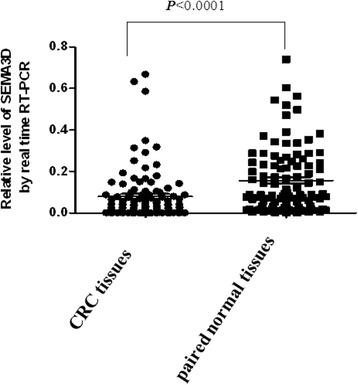
Differential mRNA expression of SEMA3D in CRC and paired normal tissues. *SEMA3D* mRNA expression was lower in CRC tissues compared with paired normal tissues (*P* < 0.0001)

Q-PCR was used to investigate whether the mRNA expression of *SEMA3D* was different in CRC tissues and paired normal tissues. As shown in Fig. 1, the expression of SEMA3D mRNA was lower in 76 of 100 CRC tissues compared with matched normal tissues. Statistical analysis revealed that the difference was statistically significant (*t* = 5.027, *P <* 0.0001).

### The serum levels of SEMA3D in CRC patients and normal healthy controls

**Fig. 2:**
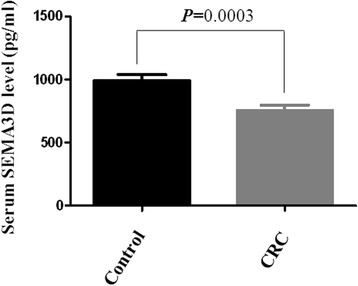
The serum levels of SEMA3D in CRC patients and normal healthy controls. Compared with normal healthy controls, the serum levels of SEMA3D were decreased significantly in CRC patients (*P* = 0.0003)

ELISA was used to measure the serum levels of SEMA3D in 80 CRC patients and 100 normal healthy controls. The results showed that the levels of SEMA3D in CRC patients were 753.23 ± 395.28 pg/ml, compared with 992.04 ± 464.97 pg/ml in normal healthy controls. The serum levels of SEMA3D were reduced significantly in CRC patients compared with controls (Fig. 2; *t* = 3.656, *P* = 0.0003).

### SEMA3D expression and clinicopathological parameters

**Fig. 3:**
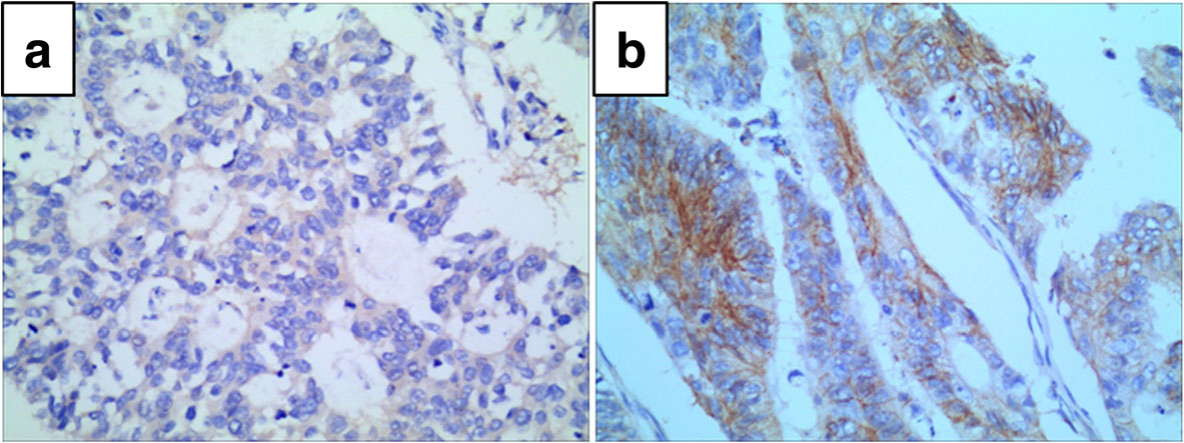
SEMA3D expression in human CRC tissues. **a** Low expression. **b** High expression. Original magnification ×400

A total of 215 CRC patients were included in the study. The low and high expression of SEMA3D, as analyzed using IHC method, is shown in Fig. 3. As shown in Table 2, there was no statistically significant association between SEMA3D protein expression and age, gender, site, size, general type, and differentiation. However, lymph node metastasis was negatively correlated with the expression of SEMA3D (*χ* ^2^ = 8.415, *P* = 0.004); patients expressing only low levels of SEMA3D were prone to lymph node metastasis.

**Table 2:** Relationship between SEMA3D expression and clinicopathologic characteristic of CRC patients

Characteristics	Number	Expression of SEMA3D		*P* value
		Low	High	
Age (years)				0.826
≤60	75	39 (52.0%)	36 (48.0%)	
>60	140	75 (53.6%)	65 (46.4%)	
Gender				0.767
Male	134	70 (52.2%)	4 (47.8%)	
Female	81	44 (54.3%)	37 (45.7%)	
Site				0.399
Colon	113	63 (55.8%)	50 (44.2%)	
Rectum	102	51 (50%)	51 (50%)	
Size (cm)				0.505
≤5	162	88 (54.3%)	74 (45.7%)	
>5	53	26 (49.1%)	27 (50.9%)	
General type				0.849
Ulcerated	93	50 (53.8%)	43 (46.2%)	
Others	122	64 (52.5%)	58 (47.5%)	
Differentiation				0.062
Well/moderate	169	84 (49.7%)	85 (50.3%)	
Poor/undifferentiated	46	30 (65.2%)	16 (34.8%)	
Lymph node metastasis				*0.004*
Negative	129	58 (45.0%)	71 (55.0%)	
Positive	86	56 (65.1%)	30 (34.9%)	

### SEMA3D expression and overall survival

**Fig. 4:**
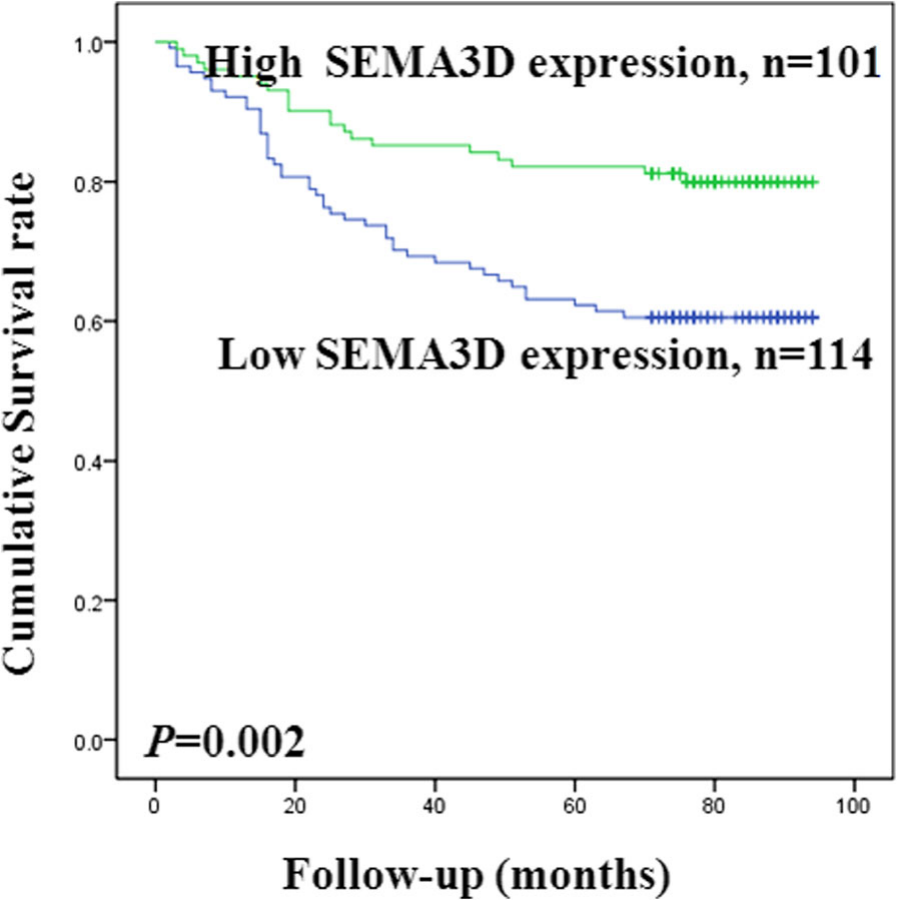
Kaplan-Meier curves in CRC patients according to SEMA3D expression. Low SEMA3D expression in CRC patients was correlated with a shorter overall survival compared with high SEMA3D expression. Log-rank tests showed statistical significance (*P* = 0.002)

All 215 patients were followed up. During the follow-up period, 65 patients died and 150 patients survived. As shown in Table 3, 45 and 20 deceased CRC patients expressed low and high levels of SEMA3D, respectively. Therefore, patients expressing low levels of SEMA3D had a shorter overall survival, whereas high SEMA3D expression indicated a longer survival (*P* = 0.002, log-rank [Mantel-Cox]; Kaplan-Meier; Table 3 and Fig. 4). High expression of SEMA3D in CRC tissues was a favorable prognostic factor. Cox’s multivariate proportional hazard model revealed that SEMA3D is an independent prognostic marker {hazard ratio [HR] 1.818, 95% CI, 1.063–3.110, *P* = 0.029) (Table 4).

**Table 3:** Univariate survival analysis of CRC

Variable	Numbers	Cases of death	*P* value
Age (years)			0.326
≤60	75	19 (25.3%)	
>60	140	46 (32.9%)	
Gender			0.706
Male	134	42 (31.3%)	
Female	81	23 (28.4%)	
Site			0.379
Colon	113	31 (27.4%)	
Rectum	102	34 (33.3%)	
Size(cm)			0.579
≤5	162	48 (29.6%)	
>5	53	17 (32.1%)	
General type			0.771
Ulcerated	93	27 (29.0%)	
Others	122	38 (31.1%)	
Differentiation			*0.016*
Well/moderate	169	46 (27.2%)	
Poor/undifferentiated	46	19 (41.3%)	
Lymph node metastasis			*<0.0001*
Negative	129	21 (16.3%)	
Positive	86	44 (51.2%)	
Expression of SEMA3D			*0.002*
Low	114	45 (39.5%)	
High	101	20 (19.8%)

**Table 4:** Results of multivariate Cox’s proportional hazard model

Variable	Relative risk (95% confidence interval)	*P*
Age	0.755 (0.442–1.291)	0.305
Gender	1.150 (0.686–1.929)	0.596
Differentiation	0.677 (0.392–1.169)	0.161
Lymph node metastasis	0.277 (0.162–0.472)	<*0.0001*
Expression of SEMA3D	1.818 (1.063–3.110)	*0.029*

### Knocking down SEMA3D promotes the migration of RKO cells

**Fig. 5:**
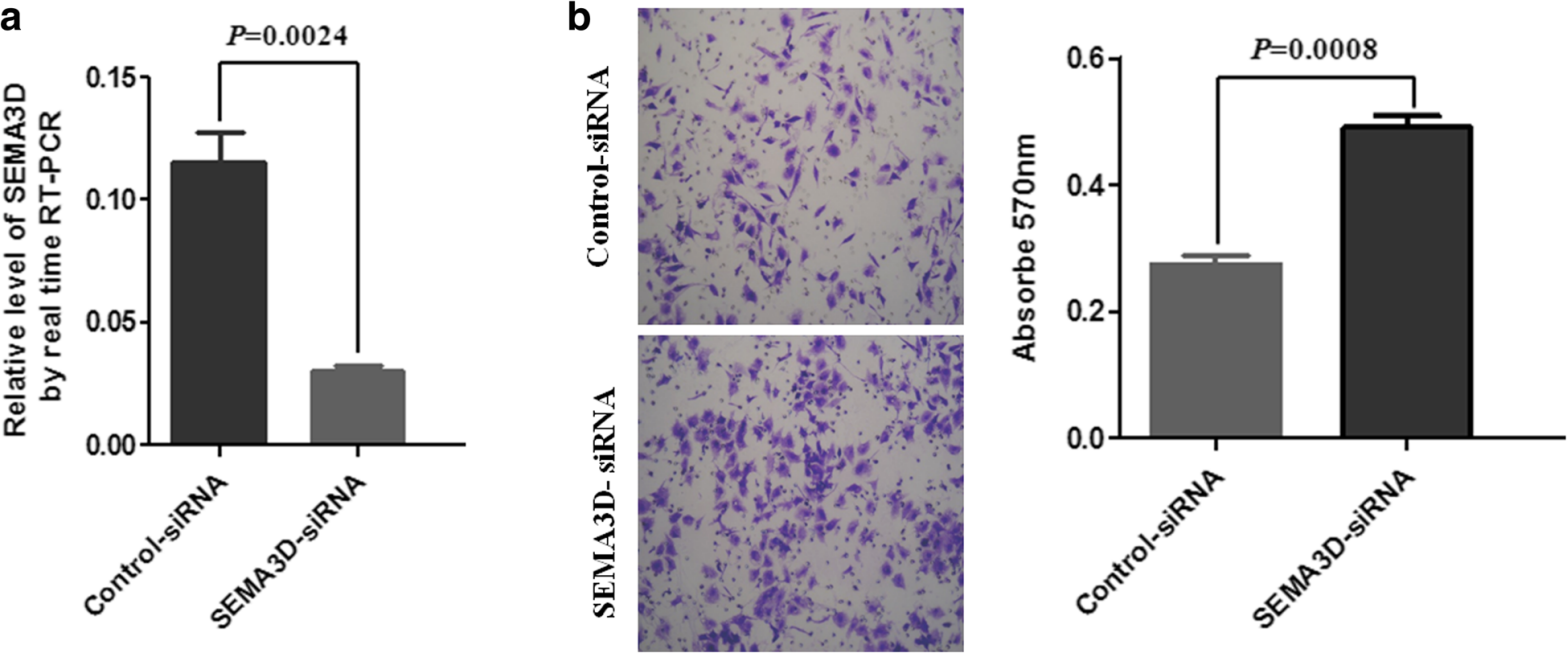
Knocking down SEMA3D promoted RKO cell migration. **a** SEMA3D was detected in RKO cells that had been transfected with control or SEMA3D siRNA using qPCR (*P* = 0.0024). **b** Migration assays were performed in RKO cells transfected with SEMA3D siRNA using transwell assays (the *right panel* shows quantification *P* = 0.0008)

Finally, siRNA was used to knock down SEMA3D in RKO cells. The results showed that the mRNA expression of SEMA3D was significantly reduced by SEMA3D-siRNA (Fig. 5a; *t* = 6.840, *P* = 0.0024). Compared with control-siRNA, SEMA3D-siRNA significantly increased the migration of RKO cells (Fig. 5b; *t* = 9.268, *P* = 0.0008).

## Discussion

Semaphorins are important axon guidance factors that play an important role in guiding the development of the nervous system [9–12]. In addition to regulating the growth of axons, semaphorins also play an important role in regulating cell proliferation and migration, as well as tumor occurrence, development, metastasis, and angiogenesis [13–21].

SEMA3D, also named coll-2 or Sema-Z2, is a secreted class-3 semaphorin. The *semaphorin* gene is located on the 7q21.11 chromosome, and it has 17 exons and 16 introns. The precursor SEMA3D peptide contains 777 amino acids, including a 36-amino acid signal peptide and a 741-amino acid mature peptide. SEMA3D is secreted into the blood, where it plays an important role. Thus, SEMA3D may be a potential serological marker for cancer.

In recent years, SEMA3D has been studied in various fields. Berndt et al. used zebrafish to demonstrate that SEMA3D could promote neural crest cell growth and proliferation via Wnt/TCF signaling [22]. However, a Japanese research group suggested that SEMA3D may damage neural development, which could be relevant in schizophrenia [23]. Ton et al. found that SEMA3D could regulate zebrafish fin regeneration via a Cx43-dependent mechanism [24]. However, another study indicated that the role of Cx43 in the regeneration of zebrafish fin needed the coordinate between SEMA3D and Hapln1a [25]. A recent study showed that SEMA3D could suppress the movement and migration of human umbilical vein epithelial cells via the PI3K/Akt signaling pathway [17]. In addition, two studies reported that SEMA3D plays a crucial role during the development of the enteric nervous system, and that abnormal SEMA3D pathway may lead to the occurrence of Hirschsprung’s disease [26, 27].

Importantly, several recent studies have investigated the role of SEMA3D in cancer. Kigel et al. demonstrated that SEMA3D can inhibit the formation of breast cancer [4]. Another study showed that the expression of SEMA3D was lower in high-grade gliomas compared with low-grade gliomas, which suggests that SEMA3D functions as a tumor suppressor in gliomas [5]. By implanting glioblastoma cells into the mouse cerebral cortex, Sabag et al. demonstrated that SEMA3D could inhibit blood vessel formation and could exert antitumor effects, which suggests that SEMA3D may be used to treat glioblastoma patients [6]. Another recent study showed that the expression of SEMA3D was low in thyroid carcinoma, and concluded that it could be used as a good diagnostic marker of cytologically indeterminate thyroid cancers [8].

In pancreatic ductal adenocarcinoma (PDA), AnxA2 can promote the secretion and thereby increase the levels of SEMA3D, and primary PDA patients that express high levels of SEMA3D have a wider range of metastases than those who express lower levels of SEMA3D [7]. SEMA3D is expected to become a novel therapeutic target and prognostic indicator for metastatic PDA. Intriguing, another study showed that human papilloma virus (HPV) can be integrated into *SEMA3D* gene introns, which promotes the development of cervical cancer [28]. These findings suggest that SEMA3D may act as an oncogene or a suppressor gene in different cancers.

Little research into the role of SEMA3D in CRC has been reported. Our previous study used mRNA microarrays to show that the expression of SEMA3D is significantly lower in CRC tissues than in paired normal tissues. In the current study, we confirmed that *SEMA3D* mRNA expression is higher in normal colorectal mucosa than in CRC tissues. These results suggest that SEMA3D may play a role in the formation and development of CRC.

The results of IHC indicated that SEMA3D was primarily localized in the cytoplasm or cytomembrane in CRC. SEMA3D expression was inversely correlated with lymph node metastasis, and patients with low SEMA3D expression were prone to lymph node metastasis. Meanwhile, knocking down SEMA3D in RKO cells promoted cell migration. These results indicate that SEMA3D may inhibit CRC metastasis. Univariate survival analysis of CRC showed that patients expressing high levels of SEMA3D had longer survival than expressing low levels of SEMA3D. This results were similar with the previous study which showed that SEMA3D can prolong the survival of the mice in glioblastoma [6]. In addition, patients with lymph node metastasis had shorter overall survival. Univariate analysis also revealed that SEMA3D expression was inversely correlated with lymph node metastasis. Moreover, a multivariate Cox’s proportional hazard model revealed that SEMA3D is also an independent prognosis marker for CRC. The above data suggest that SEMA3D functions as a protective factor in CRC.

The current study also investigated whether the serum levels of SEMA3D could be a diagnostic marker in CRC. The results suggested that SEMA3D serum levels were significantly reduced in CRC patients compared with normal healthy controls. This suggests that SEMA3D serum levels could act as a diagnostic marker of CRC.

Taken together, the current study suggests that SEMA3D may function as a tumor suppressor gene during the formation and development of CRC. It might also be a biomarker for the diagnosis, metastasis, and prognosis of CRC. In addition, the current study provided theoretical support of the mechanism for further colorectal cancer research and provided a new direction for the prevention and individualized treatment of CRC. Ultimately, this may help improve the survival quality for CRC patients.

## Conclusions

The current study indicated that SEMA3D might function as a tumor suppressor during the formation and development of CRC. SEMA3D levels were negatively correlated with lymph node metastasis. Moreover, SEMA3D was an independent prognostic marker in CRC patients. SEMA3D is a potential predictive marker for the diagnosis, metastasis, and prognosis of CRC, and it represents a novel target for the prevention and treatment of CRC.

## Abbreviations

CRC: Colorectal cancer
ELISA: Enzyme-linked immunosorbent assay
GAPDH: Glyceraldehyde-3-phosphate dehydrogenase
HPV: Human papilloma virus
HR: Hazard ratio
IARC: International Agency for Research on Cancer
IHC: Immunohistochemistry
PBS: Phosphate-buffered saline
PDA: Pancreatic ductal adenocarcinoma
qPCR: Quantitative real-time polymerase chain reaction
SEMA3D: Semaphorin 3D
siRNA: Small-interring RNA
